# Roots’ Drought Adaptive Traits in Crop Improvement

**DOI:** 10.3390/plants11172256

**Published:** 2022-08-30

**Authors:** Mirza Shoaib, Bikram P. Banerjee, Matthew Hayden, Surya Kant

**Affiliations:** 1 Agriculture Victoria, Grains Innovation Park, 110 Natimuk Road, Horsham, VIC 3400, Australia; 2 School of Applied Systems Biology, La Trobe University, Bundoora, Melbourne, VIC 3083, Australia; 3 Agriculture Victoria, AgriBio, Centre for AgriBioscience, 5 Ring Road, Bundoora, Melbourne, VIC 3083, Australia

**Keywords:** drought resilience, root exudates, root hydraulics, root ideotype, root plasticity, root shoot interaction, root system architecture, soil carbon, soil microbes

## Abstract

Drought is one of the biggest concerns in agriculture due to the projected reduction of global freshwater supply with a concurrent increase in global food demand. Roots can significantly contribute to improving drought adaptation and productivity. Plants increase water uptake by adjusting root architecture and cooperating with symbiotic soil microbes. Thus, emphasis has been given to root architectural responses and root–microbe relationships in drought-resilient crop development. However, root responses to drought adaptation are continuous and complex processes and involve additional root traits and interactions among themselves. This review comprehensively compiles and discusses several of these root traits such as structural, physiological, molecular, hydraulic, anatomical, and plasticity, which are important to consider together, with architectural changes, when developing drought resilient crop varieties. In addition, it describes the significance of root contribution in improving soil structure and water holding capacity and its implication on long-term resilience to drought. In addition, various drought adaptive root ideotypes of monocot and dicot crops are compared and proposed for given agroclimatic conditions. Overall, this review provides a broader perspective of understanding root structural, physiological, and molecular regulators, and describes the considerations for simultaneously integrating multiple traits for drought tolerance and crop improvement, under specific growing environments.

## 1. Introduction

In response to drought, roots adjust their traits, improving plant adaptation, survival, and yield. Among these traits, root system architecture (RSA) is essential in increasing water uptake [1,2], therefore, much of the research has focused on understanding RSA [3]. Phenotyping systems, such as X-ray computed tomography, magnetic resonance imaging, ground-penetrating radar, shovelomics, rhizotrons, and transparent soils, were developed to study RSA [2,4]. These phenotyping systems identified several root architectural traits that increased water uptake and drought resistance and were utilized in developing drought-resilient plants. [5,6,7,8,9]. Plants also invest a large portion of their photosynthetic carbon (C) as exudates to build root–microbe symbiosis for drought adaptation [1,2,10]. These microbes influence plants’ water uptake by extending the water harnessing area [11], changing root hydraulics [12], producing drought stress-reducing antioxidants and influencing many other physiological activities. Thus, beneficial microbial inoculants are widely used in agriculture to improve productivity. Similarly, a crucial drought response is root hydraulics, and plants regulate it for optimal water use. However, rarely it is considered together with water-efficient root architectural traits while proposing drought adaptive ideotype. The effect of interaction among multiple architectural traits and the contribution of their plasticity is also seldomly explored. A further significant response to drought is root exudation and the root-symbiotic microbes’ contribution in improving soil structure, water holding capacity, and storage and nutrient availability. However, these root soil improvement traits are yet to be fully explored for incorporation into drought-resilient crop improvement. Here, we highlight some of these significant and diverse root traits, including architecture, anatomy, hydraulics, plasticity, root-shoot interaction, exudates and interaction with microbes, and emphasize the importance of simultaneous integration of multiple traits for drought tolerance and crop improvement.

## 2. Root System Architecture and Its Interaction with the Shoot in Response to Drought

To anchor and forage through the soil for water and nutrient uptake, roots dynamically modify their spatial distribution by changing their length, depth, number, angle, diameter, density, and biomass—this spatial distribution and shape is referred as root system architecture (RSA) [2,13]. The functionality of the RSA relies on a dynamic interaction response with its heterogeneous growing environment [13], therefore, RSA has a crucial role in plant adaptation to drought [14]. Although some individual root traits have significant impact on water harnessing in drought, the efficiency and function of those traits are influenced by RSA, for example, root hairs are crucial for water uptake [15], but RSA regulates the efficiency of root hairs. Root hairs in shallow RSAs can access water from surface soil, but root hairs in deep RSAs can continue to access water from deep soil when the surface soil is dry. Roots change their RSA in response to drought, with the adaptation type varying based on the environment; for example, long and metabolically efficient RSA can access water from deep soil during drought [16]. However, in areas where water is mostly available in surface soil due to short periodic rainfall, a shallow RSA is more efficient [17]. Even within the same environment, RSA could change due to heterogeneous water distribution in soil. Roots can preferentially increase their branching towards the water, known as hydropatterning [18] and suppress lateral root formation in soil air spaces, known as xerobranching [19]. Several genes modulate RSA through gravitropic and radial growth during drought, allowing them to form shallow versus deep roots [20]. *EXO70A3* regulates the auxin pathway by acting on the distribution of the PIN4 (auxin efflux carrier), resulting in the regulation of gravitropic growth (rooting depth) in RSA [21]. Whereas CKX2 catalyzes the degradation of cytokinin at the upper lateral root flank, suppressing organ growth towards gravity, and allowing radial expansion forming shallow RSA [22].

During drought, root and shoot crosstalk and influence each other’s growth. A plant’s ability to sense drought and its intensity depends on soil moisture, temperature, radiation, and other environmental factors [23]. When roots sense water stress, it triggers changes in xylem hydraulics, mobile peptides, reactive oxygen species (ROS), and Ca^2+^ signaling, affecting shoot activities, such as stomatal closure to conserve water [24]. The plant then prioritizes its resource allocation and invests more in roots during drought [25,26], affecting the root–shoot ratio [27]. Investing in roots allows the plant to increase water uptake and maintain root water influx under drought conditions, supporting uninterrupted plant growth [24]. In white clover (*Trifolium repens*), the genotype with increased root weight in proportion to total plant weight had improved growth, survival, and seed yield during drought [28]. Chen et al. [27] reported how phosphorylation of sucrose transporters mediates the root–shoot ratio during drought, thus suggesting a strategy for developing drought-resistant crops. They reported that drought stress increases abscisic acid (ABA) levels which activates the SnRK2 protein kinases, phosphorylating the sucrose transporters SWEET11 and 12. This phosphorylation enhances the oligomerization and sucrose transport activity of SWEETs, causing elevated sucrose contents in roots, improving root growth and biomass, root–shoot ratio, and thus drought adaptation.

## 3. Roots’ Structural Response to Drought

**Figure 1:**
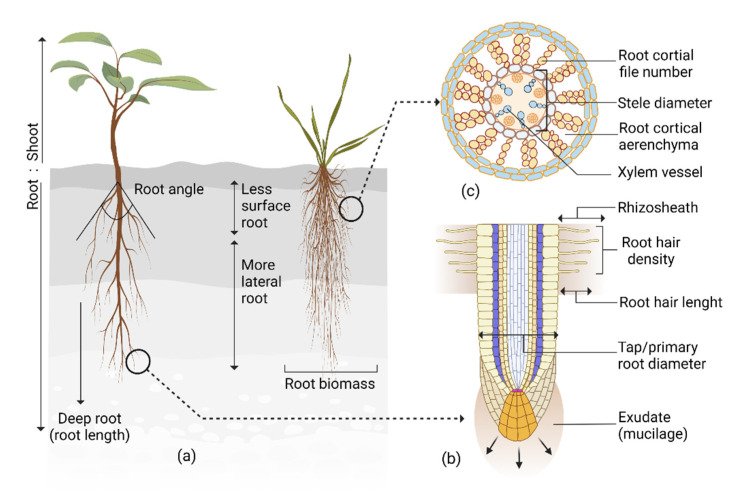
Drought adaptive root traits. (**a**) Changes in root angle, length, and biomass; the ratio with the shoot, and increased lateral branching facilitate plant adaptation to drought; (**b**) Root hair length and density, rhizosheath size, taproot diameter, and exudates are crucial drought-responsive traits. (**c**) Plants adapt their anatomical traits such as root cortical file number, cortical aerenchyma, stele diameter, and xylem vessel in response to drought.

Roots adapt their structure in response to drought to increase penetration, distribution, and contact with the soil for improved water and nutrient uptake [6,15]. These structural adaptations ensure necessary nutrition and water acquisition, maintaining plant physiological activities and productivity during drought. Several studies which reported correlations between root structural traits and plant performance under drought are summarized and presented in Table 1 and Figure 1a,b. Identifying these drought-responsive structural traits in different crops will facilitate plant breeders to utilize these traits for screening drought tolerant genotypes.

Root drought-responsive structural traits are not static but fluctuate readily in conjunction with the environment, management practice, soil microbes, and genotype. Moreover, different root traits interact, sometimes synergistically or antagonistically, affecting drought adaptation. Thus, careful consideration is needed when planning drought-resistant crops based on structural traits only. However, crop-specific drought-responsive structural traits (Table 1) are easy to phenotype and implement for screening genotypes. Therefore, it has importance in terms of ease of applicability and achieving fast results in developing drought-resistant crops.

## 4. Root Anatomical Responses to Drought

Similar to architectural traits, roots also adapt their anatomical characteristics in response to drought. Roots increase penetration in soil, reduce metabolic cost, regulate hydraulic conductivity, and facilitate microbial symbiosis to increase resource acquisition [29,30,31,32,33]. Some of these key components are described below.

### 4.1. Anatomical Adaptation in Reducing Metabolic Cost

Plants invest photosynthates in establishing root systems for exploring water and nutrients needed for metabolic processes. During drought, the expenditure of photosynthates is high, firstly to invest more in root growth to increase water uptake, and secondly to increase respiration to maintain roots in drying soil, thus, compromising plant productivity [34,35]. Anatomical adaptation during drought reduces metabolic costs and allows plants to distribute resources for further resource acquisition, growth, and essential physiological activities, resulting in improved yield. Chimungu et al. [36] reported cortical tissue with fewer but larger cell sizes, and low cortical cell file numbers in maize reduced metabolic cost by decreasing respiration rates during drought. Large cortical genotypes had increased root growth and water acquisition, deeper roots, better stomatal conductance and leaf CO_2_ assimilation, and greater shoot biomass and grain yield. Colombi et al. [34] reported a similar result in wheat, where a genotype with a large root cortical cell diameter significantly reduced root penetration metabolic cost. During drought, root cortical tissue lysed and creates an intercellular vacuum space, cortical aerenchyma [37] (Figure 1c), the formation of which can reduce metabolic costs. This mostly occurs in older roots that no longer take up water efficiently [37], thus having minimal negative impact on water uptake. Instead, it reduces soil exploration metabolic costs [35,38], permits root growth, and improves soil resource acquisition in dry conditions [35]. Under water stress, maize genotypes with high root cortical aerenchyma had 30% more shoot biomass [39].

### 4.2. Anatomical Response Improving Root Penetration

Water deficit in the soil increases mechanical impedance, thus restricting root penetration into deep soil, which hinders resource capture, consequently reducing crop productivity [40]. Anatomical adaptation facilitates improving root penetration in drying soil. For example, smaller outer cortical cells stabilize the root against ovalization, prevent collapsing, and allow the root to penetrate soil. In contrast, large mesodermis cortical cells and thick axial roots with more aerenchyma reduce the metabolic cost of soil exploration and allow root growth in hard soils [41]. In deep rooting maize genotypes, the roots generated from node three had a reduced cell file number and increased middle cortical area; while the roots generated from node four had increased aerenchyma [38]. Maize and wheat genotypes with multiseriate cortical sclerenchyma had a 22% increase in deep soil penetration and a 49% increase in shoot biomass compared to genotypes that lacked it [33]. These genotypes also had small cells with thick walls in outer cortical tissue, increased root lignin concentration, tensile strength, and root tip bending force.

### 4.3. Anatomical Attributes Facilitating Microbial Symbiosis

Roots absorb water and nutrients through the root epidermis, hairs, and avascular mycorrhizal (AM) hyphae. The AM colonize in root cortical cells and extend their hyphae into the soil, sometimes expanding soil volume exploration at least 15 cm beyond the root surface [42]. The AM receives organic C from the root and, in return, delivers nutrients to the root [43]. This mutual relationship increases drought resistance and reduces yield loss [44,45,46,47]. Root anatomical traits play an essential role in microbial symbiosis. In maize, larger root diameters and larger aerenchyma lacunae increase mycorrhizal colonization, whereas increased aerenchyma and decreased living cortical area reduce mycorrhizal colonization [31]. Cortex thickness is also crucial for AM colonization in woody species [48,49]. Dreyer et al. [50] reported that a continuous sclerenchymatic ring in the outer cortex and aerenchyma in the inner cortex decrease AM colonization in three palm species. They suggested that the sclerenchymatic ring acts as a physical barrier preventing penetration of AM fungal, while the empty aerenchyma area reduces the available tissue in the root for AM colonization.

### 4.4. Anatomical Adaptation in Regulating Water Transport 

Root anatomical traits have a substantial influence on water uptake. Through concentric layers of root cells, water first enters from the soil to the root stele, then into the xylem, and finally into the shoot [2]. Transportation in the xylem is vertical, as casparian strips limit radial movement, with the size and number of xylem vessels affecting the water transportation rate [51,52]. During root development, two types of xylem vessels are formed, the narrow protoxylem vessels and larger metaxylem vessels, through which the majority of the water is transported [51]. Prince et al. [30] reported soybean genotypes with large xylem vessels, increased xylem diameter, and metaxylem numbers performed well in water-limited environments. Large xylem vessels in olive (*Olea eurpaea*) increased root conductivity during drought stress, allowing deep rooting and extended water acquisition [53].

Phenotyping root anatomical traits is still low throughout, therefore, less research has focused on drought adaptive root anatomical traits. Further research in this area will improve phenotypic efficiency, accelerate the discovery of additional anatomical traits that assist directly in drought adaptation or indirectly through the facilitation of AM colonization, and ultimately contribute to developing drought-resilient crops.

## 5. Root Hydraulics

Root water permeability, known as root hydraulic conductivity, plays a crucial role in water uptake from soil [2,39,54,55]. Improved root hydraulic conductivity decreases the need for plants to invest in more root growth for soil exploration to increase water uptake, therefore, reducing the metabolic costs and stabilizing physiological processes under drought [30,37]. Along with root architecture, root hydraulic properties are key to predicting and interpreting plant transpiration activity during drought [56]. In water-limited conditions, root hydraulic conductivity positively correlated with dry biomass in rice and Arabidopsis [57,58]. Root hydraulics changes due to temperature [59], water availability [57,58,60], plant genotype [61,62], root anatomical traits [30,63], biochemical traits [55], and soil microbes [12,64].

**Figure 2:**
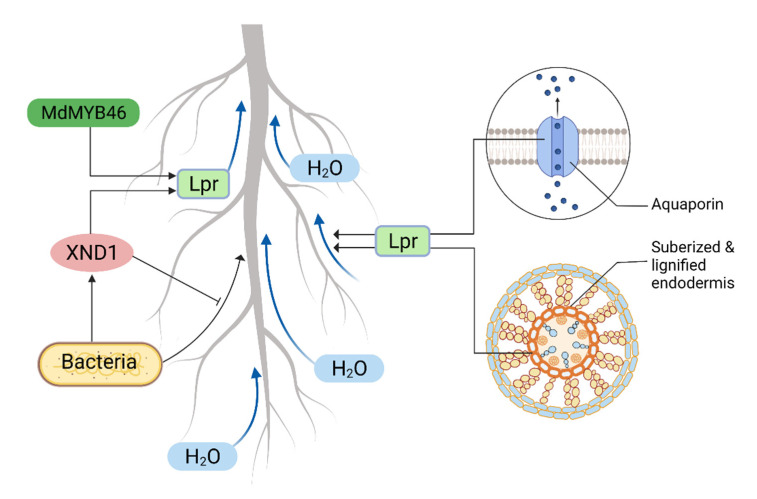
Molecular and cellular regulators of root hydraulics. *XND1* reduced root hydraulics (Lpr) by inhibiting xylem formation. Bacteria increase *XND1* activity and *XND1* reduces the pathogenicity of the bacteria; thus, Lpr is regulated without being affected by bacterial wilt. *MdMYB46* influences Lpr by modifying root xylem vessel formation. Aquaporins control Lpr by regulating radial water transport. Suberin reduces Lpr and lignin indirectly reduces Lpr by facilitating xylem vessel development.

Transcription factor *XDN1* influences root hydraulic conductivity by negatively regulating xylem differentiation (Figure 2). Loss of function of *XND1* increased root hydraulic conductivity and shoot weight, therefore, increasing drought tolerance, with opposite effects seen from the overexpression of *XND1*, which negatively regulated drought stress. *XND1* also interacts with pathogenic bacteria (*Ralstonia solanacearum*) to influence xylem formation, as the presence of the bacteria enhance the expression of *XND1*, resulting in the reduction of root hydraulic conductivity. *XND1*, on the other hand, restricts bacterial growth and thus its pathogenicity [2,57]. In apple (*Malus domestica*), *MdMYB46* was reported to regulate root xylem vessel formation, thus root hydraulic conductivity and tolerance to drought [65] (Figure 2).

Aquaporins (water channel proteins) also control root hydraulic conductivity by facilitating water diffusion across cell membranes, thus regulating radial water transport [66] (Figure 2). Root hydraulic conductivity can be increased or decreased by regulating plasma aquaporins [67,68], which can also promote the emergence of lateral roots by increasing directional water flow in the root lateral primordia [69]. During water stress, aquaporins are possibly involved in circadian oscillations of root hydraulic conductivity, facilitating root water uptake and promoting growth and photosynthesis [70].

Roots form a thicker suberized endodermis in response to drought [60]. Calvo-Polanco et al. [71] studied a collection of Arabidopsis mutants defective in suberin deposition and found mutants with enhanced root hydraulic conductivity, suggesting suberin reduces root hydraulic conductivity. Drought stress also deposits lignin in the root endodermis, indirectly reducing root hydraulic conductivity by contributing to change in xylem vessels [72] (Figure 2).

Most research has been focused on RSA, its adaptive response and role of individual RSA elements and the hormonal regulation of those elements in response to drought. However, Vadez [55] gave examples where root RSA and its element adaptive response do not fully explain plant drought adaptation and suggested root hydraulics to be considered along with RSA. Currently, several methods are available to measure root hydraulic conductivity [60,66,73,74,75]. Heymans et al. [76] proposed an inexpensive high-throughput method with some optimization suggestions. Therefore, it is feasible to phenotype root hydraulic conductivity with architectural traits.

## 6. Interaction among Drought Response Root Traits

Plants respond to various environmental conditions and stresses in the field with multiple root traits simultaneously, dynamically, and through the interaction of several traits [13]. These trait interactions sometimes have synergistic or antagonistic effects on drought adaptation [16,41]. Ajmera et al. [77] reported root trait synergism among nodal root angle, smaller diameter nodal roots, nodal root number, and L-type and S-type lateral branching densities that improve yield in dry and low nitrogen (N) conditions. Root anatomical and architectural traits can also interact synergistically, such as in common bean (*Phaseolus vulgaris*), where shallow roots with low axial conductance metaxylem improved water status by reducing water uptake [52]. This interaction of root anatomical and architectural traits likely reflected an adaptation for water uptake to their native environments. Small cells in the outer cortical region and stele diameter size can predict root penetration ability into deep soil [41]. However, these anatomical traits possibly have synergy with root growth angle and root hairs, as root angle can influence rooting depth [5], and root hairs provide anchorage to the growing root tip to penetrate drying soils [78]. Sometimes traits can interact antagonistically, for instance, forming aerenchyma tissue in the root reduces metabolic costs, improving drought adaptation [35], but this increase in aerenchyma tissue also decreases the colonization of drought-reducing root-inhabiting mycorrhiza [31,79,80,81]. The increased understanding of how plants prioritize root traits and their interaction with other environmental factors will improve the planning of ideotypes and selection of genotypes for drought-prone areas.

## 7. Root Plasticity in Drought Adaptation

**Figure 3:**
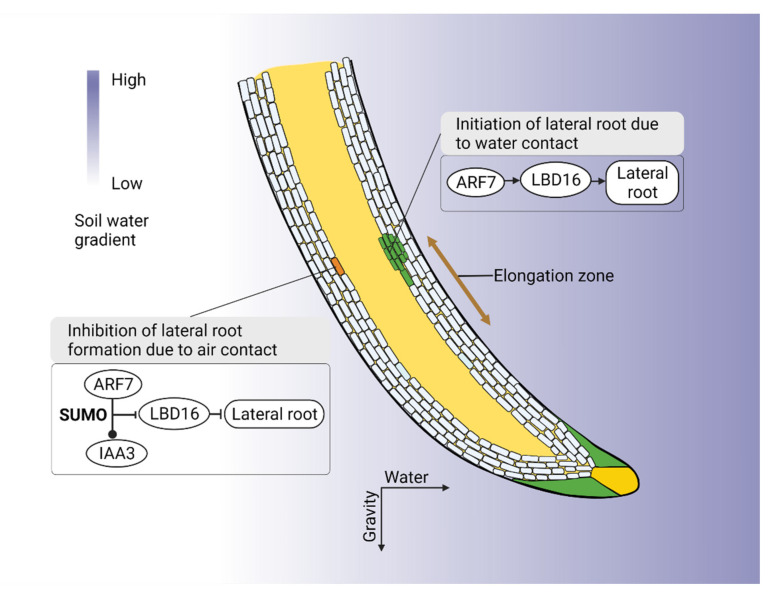
Molecular mechanism of root hydrotropism and hydropatterning. *ARF7* is crucial for preferential lateral root branching towards water (hydropatterning). *ARF7* regulates *LBD16* which initiates lateral root formation towards the water. On the dry side of the soil, SUMO protein enables *ARF7* to increase auxin repressor protein IAA3. IAA3 represses *LBD16*; thus, no lateral root formation [18,87]. In response to a water gradient, ABA mediates differential growth response. In the root elongation zone cortex, ABA signaling kinase SnRK2.2 and MIZ1 are expressed that inhibit hydrotropism by preventing cell elongation.

The adaptive abilities of roots to diverse environments, known as root plasticity, are crucial along with RSA for breeding climate resilience crops [18]. In response to drought, the *DRO1* (DEEPER ROOTING 1) gene changes rooting depth by changing root angle and increasing gravitropism, thus increasing yield [5,82,83]. The *EXOCYST70A3* gene also alters root gravitropic responses, resulting in greater rooting depth, by acting on PIN4 protein distribution, influencing auxin transport [21]. During drought, where water is available deep in the soil, fewer but longer lateral roots in conjunction with deep rooting reduces root respiration and improve biomass and yield [6]. Conversely, in low periodic rainfall areas where water is available mainly in surface areas, more lateral branching towards the surface improves water harnessing efficiency during drought [84]. Plasticity in lateral root formation towards available water i.e., hydropatterning, is therefore crucial in drought adaptation. In Arabidopsis, *LBD/ASL* genes are responsible for lateral root formation with AUXIN RESPONSE FACTORs, *ARF7* and *ARF19*, directly regulating these genes [85,86]. Orosa-Puente et al. [87] found roots preferential lateral root branching towards water depends on auxin response factor *ARF7*, inducing differential expression of *LBD16* in the lateral root, with *ARF7* expression itself regulated by the small ubiquitin-like modifier (SUMO) proteins. Arabidopsis plants lacking the SUMO protein had defective hydropatterning similar to arf7 mutants [18,87]. In wheat, the lateral root number is regulated by *TaLBD16-D* [88], while in rice (*Oryza sativa* L.), auxin transporter, *OsAUX1* controls lateral root initiation [89] (Figure 3).

Hydrotropism (the growth of plant roots towards moisture) also plays a key role in drought adaptation. In response to heterogeneous low water potentials, root tips overcome gravity-driven growth and increase branching towards higher water regions, i.e., hydropatterning [90]. When emerged lateral roots are surrounded by dry soil, hydrotropic growth assists roots to maneuver toward the water [18]. Thus, hydrotropism and hydropatterning interplay to adapt plants in a drought. Hydrotropism is dependent on ABA and independent of auxin redistribution, however, its molecular and cellular basis remains unclear [90,91]. Earlier, Kobayashi et al. [92] suggested the hydrotropism response is independent of gravitropism. Recently Dietrich et al. [91] showed the meristem at the root cap does not direct hydrotropism; rather, it depends on the root elongation zone’s SnRK2.2 (ABA signaling kinase) and MIZ1. They suggested that the root elongation zone influences hydrotropism by sensing a water potential gradient and undergoing differential growth (Figure 3).

## 8. Root Exudates and Microbial Symbiosis in Drought Adaptation

Plants release exudates and root litter into the soil, which microbes feed on and decompose, enhancing nutrient availability in the soil and promoting root growth. Through this symbiotic relationship, root exudates and root-associated microbes can improve plant resilience and performance under drought [93,94]. Out of these microbes, AM and rhizobacteria are major contributors to drought resistance. Here, we highlight a few examples of their contribution to plant drought adaptation.

### 8.1. Root Exudate Role in Drought Adaptation

**Figure 4:**
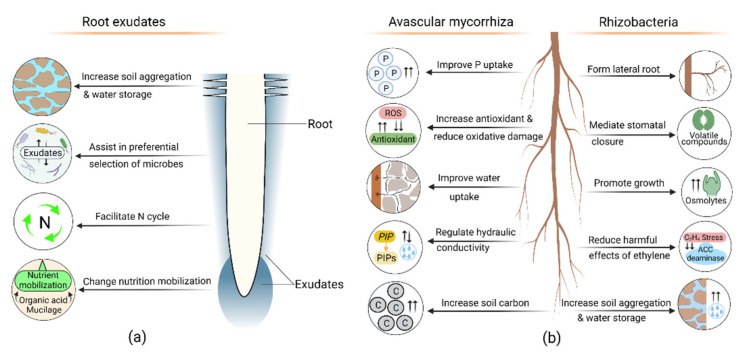
Beneficial roles of root exudates, avascular mycorrhiza (AM), and rhizobacteria in plant drought adaptation. (**a**) Exudates effects soil aggregation, water holding capacity, and nutrient mobilization. The plant preferentially selects microbes through exudation, which assists in drought adaptation. Exudates also influence the soil nitrogen (N) cycle. (**b**) AM increases phosphorus (P) and water uptake, affecting root hydraulic conductivity and reducing drought stress by producing antioxidants. AM also increases soil carbon. Rhizobacteria release exopolysaccharides, volatile compounds, osmolytes, ACC-deaminase, and phytohormones. These compounds increase soil aggregation, lateral root formation, and plant growth; mediate stomatal closure, reduce ethylene’s harmful effect, and ultimately increase drought resistance.

Plants change their exudate composition during drought, impacting the plant itself, neighboring plants, soil properties, and organisms [95]. Exudates also serve as a chemoattractant and nutrition source for many drought-reducing microorganisms. For instance, during drought, plants increase their mucilage production, resulting in a larger rhizosheath [96]. Root mucilage plays an important role in retaining water; thus, large rhizosheath formation increases the plant’s adaptation to drought [96,97,98]. Plants preferentially recruit root colonizing drought-reducing microbes by adjusting the composition of root exudates [99,100,101,102]. For instance, during drought, plants produce Glycerol-3-phosphate, which increases the abundance of drought stress-reducing monoderm bacteria [103], while maize increases organic acid exudation, particularly malic acid [104], attracting *Bacillus subtilis* known to aid plant drought resistance [105]. Naylor et al. [106] found different plants prefer a specific drought-stress reducing Actinobacteria community, suggesting the plants favored a particular group of microbes for drought adaptation. During drought, exudates including organic acids and mucilage improve nutrition mobilization [107,108], as well as facilitate the belowground N cycle, influencing iron uptake and indirectly assisting in drought adaptation [10] (Figure 4a).

### 8.2. Contribution of Avascular Mycorrhizal Symbiosis in Drought Response

Avascular mycorrhiza can increase drought resistance and reduce drought susceptibility [109] by improving water and P uptake and enhancing photosynthetic performance [79]. Additionally, through extraradical hyphae, AM extends the soil volume connection beyond the root zone, thus, increasing water uptake [11,110]. AM can occasionally modulate lateral root formation such as in lemon (*Citrus limon* L.) [111] and maize [112]. Symbiosis of AM can influence root hydraulic properties and enhance plant drought resistance by controlling the *PIP* gene that regulates plasma-membrane proteins (PIPs). The downregulation of PIPs reduces water loss during drought [113]. Aroca et al. [12] found AM symbiosis in *Phaseolus vulgaris* was strongly correlated with the regulation of PIP2 protein and reduced hydraulic conductivity during drought. In *Ulmus americana*, AM increased apoplastic water transport and root hydraulic conductivity [64]. During drought, ROS production causes oxidative damage, degrading pigments, proteins, lipids, carbohydrates, and DNA [114]. However, the enzymatic components—superoxide dismutase, catalase, ascorbate peroxidase, glutathione reductase—and non-enzymatic components—cysteine, glutathione, and ascorbic acid—of exudates work as antioxidants and prevent this oxidative damage [114]. AM can increase antioxidant enzyme activities, thus reducing oxidative stress during drought [81], such as inducing more hydrogen peroxide effluxes of the taproot and lateral root of oranges (*Poncirus trifoliata* L.) [80]. In addition, AM contributes to improved soil C [115], which affects water storage, thus indirectly contributing to increased drought resilience (Figure 4b).

### 8.3. Role of Rhizobacteria in Drought Adaptation

Rhizobacteria release phytohormone, for example, IAA, which promotes lateral root formation [116]. Rhizobacteria inoculated grapevines had increased IAA content and drought resistance [117]. Rhizobacterial exopolysaccharides mediate soil water content, increase soil aggregation, and form a protective capsule around soil aggregates [118], creating an area where water remains longer than the surrounding area [119]. Due to this increased soil aggregation and water content permeability, plants have improved water and nutrient uptake during drought [118]. Some rhizobacteria release volatile compounds that can increase drought resistance in the plant directly and/or indirectly [120], for example, volatile compound acetic acid stimulates bacterial biofilm formation and exopolysaccharides, a major biofilm constituent that increases water retention [120,121]. Similarly, Rhizobacteria, *Pseudomonas chlororaphis* O6, produced volatile metabolites, 2R, 3R-butanediol, mediating stomatal closure [122]. Water deficits increase the concentrations of C-rich compounds that adjust metabolic activity and promote growth, many of which are osmolytes [123,124]. In response to drought, rhizobacteria also produce osmolytes which act as a substitute for plant-produced osmolytes [125]. Staudinger et al. [123] found Rhizobia inoculated *Medicago truncatula* had an enhanced concentration of osmolytes, resulting in a delay in drought-induced leaf senescence. Under stress, ethylene regulates defense and growth responses, reducing root and shoot growth [124,126], with 1-aminocyclopropane-1-carboxylate (ACC) a crucial component of ethylene biosynthesis [126]. Rhizobacterial ACC-deaminase segregates and degrades plant ACC, thus interrupting ethylene biosynthesis and reducing the harmful effect of ethylene in drought [127]. During drought, rhizobacteria also increases activities of the antioxidant enzymes in faba bean (*Vicia faba*) [128] pea (*Pisum sativum* L.) and tomato (*Solanum lycopersicum* L.) [129,130]. Rhizobacteria similarly assist lateral root formation, thus increasing water uptake in maize [131] (Figure 4b).

Although some advances have been made in understanding the role of exudates and microbe symbiosis in plant adaptation to drought under a controlled environment, the study of root exudation in a natural setting is in its infancy [94]. Therefore, it requires a practical standardized root exudates collection method applicable in the field condition [94,132]. To increase the understanding of exudate function and microbial interaction during drought requires how; (i) intensity and composition of exudate changes in response to neighboring plants, (ii) root exudate components interact in the soil, (iii) microbes influence plant exudation, and (iv) microbial mucilage and hyphal exudation influence the overall process.

## 9. Root Soil Building Attributes in Drought Adaptation

Root and soil interchangeably alter each other, heterogeneous soil can change RSA, similarly root change, and improve soil [133]. Soil structure, nutrient availability, water dynamics, and C storage can be significantly altered by root, improving plant adaptation to drought. Thus, these root soil-building attributes contribute considerably to drought-tolerant crop improvement.

**Figure 5:**
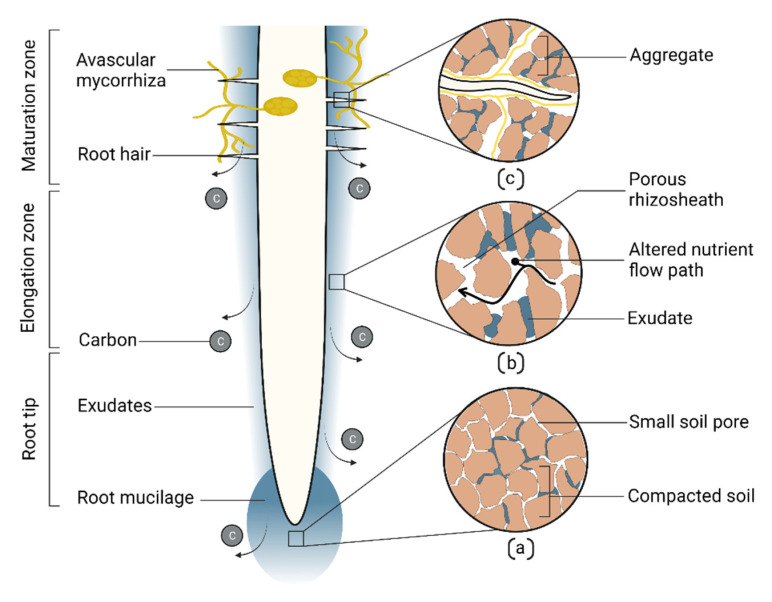
Root soil building attributes. Root exudation releases carbon into the soil. (**a**) Soil compaction and reduction of pore space in the root tip area. (**b**) Formation of porous rhizosheath area and altered nutrient flow path due to root mucilage. (**c**) Soil aggregation by root mucilage, root hair, and avascular mycorrhiza hyphae.

Roots change soil structure through compaction [134], dispersion [7], aggregation and creation of biopores [135], acting as a biological tiller [136]. When the root enters the soil, it first compresses the soil [133,134], then as the root grows, it releases mucilage and exudates, creating a porous rhizosheath [7,137] (Figure 5a). As the root grows further, its elongation zone transits into maturation, producing root hair, releasing exudates, and contributing to soil aggregation [138] (Figure 5c). Similarly, root symbiotic Rhizobacterial exopolysaccharides, AM hyphae, and hyphal exudates increase soil aggregation and change in soil structure [118,139]. Roots also improve soil structure by depositing a substantial portion of photosynthetic C as exudates (Figure 5). Below ground C assimilated by annual crops and grasses accounts for 21% and 33%, respectively [140]. This C increases water sorptivity and storage, and changes nutrition transport, ultimately improving drought adaptation. Moreover, roots represent a large proportion of the world’s vegetation C pool [141], contributing more C to the soil. Among these root carbons, deep root carbon is more stable as C decomposition is limited in deep soil [142]. Lignin and suberin in the root cell wall are also key components stabilizing soil C as these degrade slowly [40,142]. Exudate supports rhizobiome communities that release additional C in the soil [143]; thus, roots influence overall C composition, flow, and soil structure near the root zone [144].

The root rhizosphere influences spatial heterogeneities and changes the hydrophilic nature of soil [7,137,145], increasing soil porosity and rendering the rhizosphere anisotropy. It contains a significantly higher water content than bulk soil, with drought-resistant genotypes generating a larger and more porous mass of rhizosheath than drought-sensitive genotypes [7]. Greater mucilage in drought-resistant genotypes may be the main reason for a porous rhizosheath and increased water storage. These changes in the soil are later shaped by rhizosphere microbes [7,145] and create further spatial heterogeneities in the soil, such as Rhizobacterial exopolysaccharides increasing water storage length in the root zone [119]. Root exudates also regulate nutrition flows in soil, with increased exudate concentration blocking soil pores, increasing the friction of immobile water, and creating slow-conducting flow paths, resulting in changes in nutrition transport in the soil [108]. Roots can affect nutrient availability in the soil according to plant needs, for example, organic acids in root exudates act as chelating agents, resulting in increased nutrient availability for ions such as iron and P [146].

Due to current cultivation practices, soil C is reduced in agricultural land, therefore affecting the water storage capacity. Currently, the global C debt in agricultural land for the top two meters of soil is 133 petagram [147]. Drought will continue to worsen due to reduced freshwater supply, soil water absorption and capacity. Roots and their symbiotic microbes offer huge potential for mitigating atmospheric C by sequestering it in the soil. All these roots’ direct and indirect soil-building attributes can significantly increase resilience to drought.

## 10. Drought Adaptive Root Ideotypes

Proposing an ideotype for drought defines idealized approaches for a breeding program to work towards. A universal ideotype for drought in a broader range of crops might not be applicable for a specific crop because roots of each crop interact and respond differently in response to soil type [148] and growing environment. However, several root drought-resistant traits are shared in a broad range of crops.

**Figure 6:**
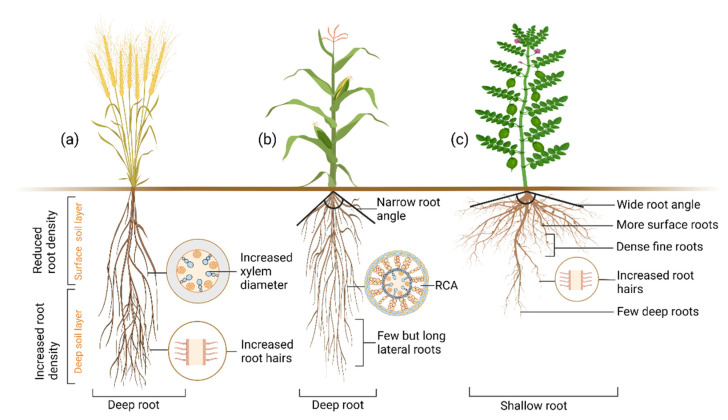
Different drought-adaptive root ideotypes. (**a**) Deep root, reduced root on soil surface layer, more root in the deep layer, and increased root hair and xylem diameter are crucial drought adaptation response traits (**b**) Deep root, narrow root angle, few but long lateral roots and more root cortical aerenchyma (RCA) are helpful for drought adaptation. (**c**) Shallow, wide-angle roots, more dense surface roots but few deep roots, and increased root hairs are the necessary root drought adaptation traits for low rainfall areas.

Wasson et al. [149] proposed deep root systems, increased root length density in medium and deep soil layers, reduced root length density in the topsoil, increased root hair growth, and increased xylem diameter as an ideal drought-resistant ideotype (Figure 6a). The deep root system would allow access to subsoil moisture in dry conditions but comes at the cost of C, otherwise, the plant would invest more in shoot and reproductive organs. However, it is possible to increase the rooting depth without extra C input by modifying specific root lengths [149]. During anthesis, insufficient root length density at depth can cause a lack of access to water in deep soil. Wasson et al. [149] proposed an ideal ideotype for drought tolerance, with few roots at the surface and more roots at depth, which have a large xylem and increased root hairs. Larger xylem lowers axial resistance, allowing water to pass from the soil into the root easier, and increased root hairs maintain higher hydraulic continuity between roots and soil, improving water acquisition in drying soil. Lynch [16] proposed a “steep, cheap, and deep” ideotype for efficient moisture uptake from the subsoil (Figure 6b), where cheap traits are mostly anatomical traits that reduce the metabolic cost of soil exploration (some of these traits are described in the above sections). Interestingly, Rao et al. [17] argued that the “steep, cheap, and deep” ideotype might not be efficient in low rainfall temperate regions with little water in deep soil, and dicot pulse crops might respond differently to monocot cereals. Thus, their “wide, shallow, and fine” ideotype emphasized dense roots in the upper surface to capture water from low rainfall events before it is lost by evaporation (Figure 6c). These shallow, fine roots might have an advantage in capturing immobile nutrients [84], such as P. How these attributes might increase drought adaptation need to be investigated to quantify the merits of the “wide, shallow, and fine” ideotype alone. In addition, further research is needed to understand the effect of deep roots on (i) biological tillage [136]; (ii) biopores creation [135]; and (iii) C input in soil, and its impacts on soil water storage, subsequent crops, and long-term drought resilience. Perhaps, the immediate short-term solutions to drought resistance are in the “wide, shallow, and fine” ideotypes and long-term solutions are the deep root ideotypes.

Contrasting root architectures—deep [16] versus shallow [17]—are proposed for drought-resistant ideotypes for different environments, thus, indicating the vulnerability of the ‘drought ideotype’, which is heavily biased by the plant growing environment. In addition, root architecture and growth do not fully represent root water uptake capability, and more profuse root growth does not necessarily relate to more water extraction [2,55], which also depends on the root hydraulics [2]. Thus, capturing the root’s complete water-harnessing ability and increasing the root drought adaptive ideotype reliability requires the consideration of root hydraulics along with architecture.

The success of a drought-resistant ideotype for a cropping system will depend on genotype, environment, and management practices. Formulating a root ideotype that performs well in broader and varied areas will require incorporating multiple drought response traits. Moreover, developing plants based on a particular root ideotype for a large area will reduce the root diversity and might lack the necessary adaptive ability to cope with climate change. Thus, a root ideotype targeted for a small and specific area might be efficient and facilitate root diversity. When formulating ideotypes, we should also consider incorporating sustainability and soil regeneration along with maximizing productivity. Incorporating root soil-building attributes in the drought ideotype offers a scope of soil regeneration and long-term resilience to drought.

## 11. Conclusions

Roots are the other half of plants that can significantly contribute to sustain productivity under drought conditions. A range of root attributes, namely RSA, root structural, anatomical, and hydraulic traits are important for drought adaptation. Equally, interactions among these traits, their plasticity and root soil building attributes also affect the overall adaption process. Research has largely been focused on root architectural responses when developing drought-resilient plants. However, root structural, physiological, and cellular regulators, and their interactions with microbes, are also important. Several root structural traits, such as taproot diameter and length, root hair number, density and length, and root angle are phenotyping-friendly, high-throughput, and relatively easy to deploy in plant breeding. Whereas, root anatomical trait screening is still low throughput, requiring further research to find traits that improve root penetration in dry soil, reduce metabolic cost, improve water uptake, and facilitate AM colonization. Consideration also needs to be taken to incorporate root hydraulics and associated molecular regulators such as *XDN1*, *MdMYB46*, and aquaporins (Figure 2) to ensure increased water uptake. Molecular mechanisms for root plasticity, hydropatterning, hydrotropism, and gravitropism are well characterized (Figure 3) to harness this knowledge in drought-resilient crop improvement.

Improvements in root exudate collection and rhizosphere-microbe phenotyping methods will increase understanding of plant–microbes responses during drought. Improving soil C can significantly augment water absorption and storage and offers potential for a long-term solution to continue increasing agricultural production under a limited water supply. Overall, careful selection and incorporation of root architectural, structural, anatomical, hydraulic, molecular, and soil building traits, and consideration of root trait plasticity and interaction are integral in developing desirable ideotypes to sustain agricultural production in drought-prone areas.

## Figures and Tables

**Table 1:** Root structural traits and their adaptive response to drought.

Structural Root Traits	Drought Adaptive Responses	Crop	Reference
Taproot diameter	Large taproot diameter genotypes had increased yield and drought resistance.	White clover (*Trifolium repens* L.), Soybean (*Glycine max* L.), Chickpea (*Cicer arietinum* L.)	Caradus and Woodfield [28], Fenta et al. [29], Rabbi et al. [7]
Taproot length	Long taproot genotypes yielded higher.	Soybean (*Glycine max* L.)	Jumrani and Bhatia [30]
Root hair	Reduced root hair genotype had lower water absorption and decreased drought resistance.	Arabidopsis (*Arabidopsis thaliana* L.)	Tanaka et al. [31]
Root hair production time	Drought-resistant genotypes had faster root hair production.	Barley (*Hordeum vulgare* L.)	Carter et al. [32]
Root hair length and number	Longer and higher root hair genotypes had less negative leaf water potential and improved water status under drought.	Barley (*Hordeum vulgare* L.)	Marin et al. [33]
Rhizosheath size	Large rhizosheath genotypes were drought resistant. Longer and denser root hairs contributed to larger rhizosheath formation.	Barley (*Hordeum vulgare* L.), Lotus (*Lotus japonicus* L.), and Maize (*Zea mays* L.)	Liu et al. [34], Rabbi et al. [7].
Root growth angle and rooting depth	Narrow root angles had downward root growth resulting in deep rooting and better yield under drought.	Rice (*Oryza sativa* L.), Soybean (*Glycine max* L.)	Uga et al. [5], Gobu et al. [35], Fenta et al. [29]
Seminal and nodal root angle	Steeper seminal and nodal root angle genotypes had a higher yield.	Maize (*Zea mays* L.)	Ali et al. [36]
Tap and lateral root branching intensity	Drought-resistance genotypes had more tap and lateral root branches.	Soybean *Glycine max* L.)	Fenta et al. [29]
Number of crown root	Low crown root number genotypes had better water status and yield.	Maize (*Zea mays* L.)	Gao and Lynch [8]
Quantity of fine-diameter roots	Drought-resistant genotypes had substantial amounts of small-diameter roots in deep soil.	Wheat (*Triticum aestivum*)	Becker et al. [25]
Lateral root branching density	Genotypes with fewer but longer lateral roots had better water status, biomass, and yield.	Maize (*Zea mays* L.)	Zhan et al. [6]
Root length, branching rate and surface area	Drought-resistant genotypes had increased root length, branching rate, larger root surface, and decreased coarse to fine root ratio.	Oat (*Avena sativa* L.)	Canales et al. [37]
Root volume and dry matter	Drought-resistant genotypes had larger root volumes and more root dry weight.	Sorghum (*Sorghum bicolor* L. Moench)	Kiran et al. [9]

## Data Availability

Not applicable.
